# Dr. Abercrombie on Stricture of the Sigmoid Flexure of Colon Case of Suspicious Hysteria

**Published:** 1830-07-01

**Authors:** 


### STRICTURE OF THE SIGMOID FLEXURE OF THE COLON.*

Two well-marked cases of this terrible disease, supervening on an acute affection of the mucous membrane of the intestines, are related in the new edition of Dr. Abercrombie's work upon the stomach.

*Case* 1. A gentleman, æt. 30, who had suffered long from pectoral complaints and scrofulous sores, was attacked in December, 1828, with symptoms of inflammation in his bowels, for which he was actively treated by Dr. Ballingall. The urgent symptoms subsided in eight or ten days, but a constant uneasiness about the pubes remained, which was not relieved by gentle laxative medicines. These operated readily, but the motions were in general scanty and occasionally he had frequent calls with mucous discharges; the appearance was unhealthy; the appetite bad, with debility, gradual emaciation, and considerable urinary irritation. When seen by Dr. Abercrombie in April, 1829, the abdomen was free from distention, and no organic disease could be discovered, but the bowels were irregular, being sometimes confined, and sometimes rather irritated with frequent slimy discharges. He continued to emaciate progressively till about the 26th of May, when suddenly his bowels became invincibly obstructed, and the abdomen grew distended, tense and tympanitic. He survived seven days in this condition with very little vomiting, and died exhausted on the 3d of June.

*Sectio Cadaveris.* "The peritoneal cavity was distended with gas, and also contained an immense quantity of fluid feces. On the surface of the intestines there was a tinge of recent peritonitis. The small intestines were moderately distended; the colon appeared to have been in a state of extreme distention; but it had burst at the caput coli by an irregular opening, and had fallen together without contraction. At the bend of the sigmoid flexure next the rectum, the intestine formed a hard mass about two inches in length, and the calibre of the canal, as it passed through this part, was contracted to a space which only transmitted a full-sized catheter. The contraction was occasioned by a uniform thickening of the parietes at the part; they were of scirrhous hardness, and the internal surface had an ash-colour and an irregular tubercular aspect. The portion thus affected was about two inches in extent, and the intestine immediately above and below was entirely healthy."

*Case* 2. A lady, ætat. 63, had been liable for several years to a confined and flatulent state of her bowels. In June, 1829, she had a violent attack of pain in the abdomen with hiccup which continued for several days. In July she had diarrhœa, succeeded by another attack of violent pain, which was followed by several evacuations, consisting chiefly of blood. From this time the bowels continued very irregular until

---

* Abercrombie on the Stomach, &c. 2d Edition, 1830.

August, when, after a severe attack of diarrhœa of several days' duration, she was seized with severe pain, followed by tumefaction of the abdomen, small rapid pulse, and prostration. The pain now recurred in paroxysms with intense severity, and there was occasional vomiting. The bowels, which at first were moved with difficulty, after some time became totally obstructed. She died exhausted about three weeks from the commencement of this attack, and a week from the time when the total obstruction of bowels took place.

*Sectio Cadaveris.* "The intestines were in a state of extreme distention, especially the colon, which was enormously distended, from the caput coli to the sigmoid flexure. It then became abruptly contracted, and at this place a stricture was found, by which the canal of the intestine was so contracted as scarcely to admit the point of the blowpipe. The part was of nearly cartilaginous hardness, and was covered by irregular scirrhous indurations. The intestine below the stricture was collapsed and healthy."

From the cases of contraction of the sigmoid flexure of the colon which we have witnessed we should say that its ordinary history differs from that of the foregoing cases. The patient, for the most part, complains of a costiveness gradually growing upon him, for which he has resorted to purge after purge, with temporary benefit from each new drug, but a speedy experience of its inefficiency. The intervals between the stools become longer and longer, the belly is blown up with intestinal tympanitis, the patient has a fæcal taste in his mouth and vomits nearly all he takes into his stomach whether it be food or medicine, his body exhales a sickly cadaverous or even fæcal odour, and at length he either sinks from the incessant irritation of the pent up fæces, or is cut off by an attack of peritoneal inflammation. Of the medicinal treatment we need not speak, but it is difficult to know what to say to the proposal of establishing an artificial anus in the other groin. Supposing that the operation succeeded, a very unlikely supposition, would not the loathsome remedy be worse than death? And besides, if the excrement were voided from the groin, yet the contraction of the colon is generally scirrhous or malignant in its nature, and would probably, were time allowed, go on to the destruction of the individual by ulceration and its consequences.

### II. Alarming Symptoms from Distention of the Intestines.

Those who see much of practice, and consequently those who see much of hysteria, may perchance be tempted to imagine that they recognize an old acquaintance with a new name in the following case. We shall give it, for it is brief, in the words of Dr. Abercrombie.

*Case.* "A lady, aged 23, had been long affected with pain in the right hypochondrium, and a very confined state of the bowels, for which a great variety of treatment was adopted with little benefit. In the autumn of 1822, the abdomen became greatly enlarged, tense, and painful. Some relief was obtained from topical bleeding, blistering, and purgatives; but after a severe pulmonary attack in winter, the pain and weight were aggravated, and extended into the left side in the direction of the arch of the colon, with increased tenderness of the abdomen. In spring 1823 she was somewhat improved, but in June and July there was again an increase of the abdominal pain, which became very severe in the course of the transverse colon, with obstinate costiveness, dry tongue and thirst. Some relief was again obtained from topical bleeding, purgatives, and enemata; the latter bringing off frothy discharges, and much flatus. In the beginning of winter 1823-4, she had two pulmonary attacks, after which the abdomen became again very tumid and painful. In April, 1824, she had pain in the right shoulder, pain and numbness of the right thigh and leg, and she often comcomplained of a feeling as if scalding water were passing along her right side. In June, the abdominal pain and tension being very great, a caustic issue was inserted on the right side of the linea alba; purgatives were persevered in; and she went to the country, where she remained during the summer and autumn, and improved considerably in strength. From this time her complaints continued to abate, and she has since enjoyed very tolerable health. The uterine functions had been through the whole course of this affection, quite natural."

We do not affirm that this was wholly and solely a case of hysteria, but we contend that it was very like it, and are sure that we have witnessed hysterical cases so closely resembling it in many respects, that it would sorely puzzle a keen nosologist to distinguish and define the points of difference. A young woman affected with obstinate costiveness, pain in the *right hypochondrium*, tumid belly, and various odd sensations, is a patient who, in nine cases out of ten, would be treated with success by purgatives, blisters, and steel, or the antispasmodic medicines. Such a case is one that is constantly occurring to the man in practice, which admits of much benefit from judicious measures, and will certainly be aggravated by extravagant or bold depletion. We may be told that the uterine functions were natural, but that does not materially affect our statements, for it is not uncommon to see females affected with most of the symptoms of chlorosis, or all the extravagancies of hysteria, whose catamenia continue regularly during the time. We have said that this is the case for blisters, and the more we see of this mode of practice in hysterical pains, the more we are convinced of its utility. The remedy acts in two ways; in the first place it is disagreeable—an excellent qualification; and in the second it unloads the vessels of the part of that congestion or plethora, call it what we will, that frequently obtains under these circumstances. But we cannot enter on the subject of hysteria, a subject that requires more time and space than we can possibly devote to it here. It is fair, however, to Dr. Abercrombie to add his remarks to the case on which we have been commenting.

"A sister of this lady was affected in a similar manner, suffering most intense pain in the abdomen, and such tumefaction that she was supposed to have ascites, and was several times on the point of being tapped. She died after protracted suffering, which continued for several years; and, on examination, the disease was found to consist entirely of an enlargement of the colon. A portion of it 44 inches in length is preserved; the largest circumference of which is 25 inches, the smallest 16. It was in many parts ulcerated.

"The existence of ulceration in this case gives reason to believe that the disease was originally connected with inflammatory action of a low chronic kind, which gradually destroyed the natural action of the part. But without any cause that can be traced of this nature, there appears to be a disease of the intestinal canal depending upon a gradual loss of its muscular power, the cause of which eludes our researches. An interesting example is related by Dr. Parry, in the case of a medical gentleman who had been long liable to dyspeptic complaints, great flatulence, and irregularity of his bowels. After suffering, for a fortnight, pain in the bowels, with nausea and costiveness, he was seized with symptoms of ileus, accompanied with severe pain, which was most violent in the epigastrium and left hypochondrium. Under the usual treatment this attack subsided after several days; but he continued from this time to be liable to similar attacks, which were accompanied by vomiting, obstinate costiveness, and severe pain, with hardness and distention in the epigastric and left hypochondriac regions. The bowels were at all times unmanageable, and the motions thin, scanty, and not formed. The pulse was little affected. The matter vomited at length became feculent, and he died with symptoms of peritoneal inflammation, about six months after the commencement of these attacks. On inspection there were found marks of peritonitis with adhesions; and the omentum was in a thickened and hardened condition. But the principal appearance was an enormous and uniform distention of the colon, the arch of which occupied entirely the epigastric and hypochondriac regions, so that the stomach and the liver were pressed upwards, high into the thorax. Its coats were in some places slightly thickened, and the peritoneum covering it was of a dark colour, but there was no appearance of contraction or obstruction in any part of its course. The enormous distention extended from its commencement to the sigmoid flexure, and it contained an immense quantity of feculent matter, partly solid and partly fluid. The sigmoid flexure and rectum were perfectly healthy. The ileum was distended, and dark coloured, but in a much less degree than the colon."

Dr. Abercrombie gives a striking example of the powers of galvanism in some obstinate affections of the bowels. A gentleman had been under the care of the most eminent physicians in England and Ireland for an obstinate state of the bowels, originally ascribed to having slept in a newly-painted room. He emaciated much, and the complaint had now gone on for two years, when Dr. Cheyne recommended galvanism. In about three weeks the natural action of the bowels was restored, and he soon recovered perfect health.

